# Impact of COVID-19 on Medical Students’ Learning Behaviours, Clinical Training, Performance as Future Medical Professionals, and Perceived Stress: A Cross-Sectional Web Survey

**DOI:** 10.7759/cureus.81540

**Published:** 2025-03-31

**Authors:** Bidyadhar Sa, Reisha N Rafeek, Asilah Ali, Aviel Smith, Avonell Wickham, Bakhita Johnston, Betricia Stowe, Brandon Cross, Brandon Seenath, Brent Rajkumar, Terence Seemungal

**Affiliations:** 1 Centre for Medical Sciences Education, Faculty of Medical Sciences, The University of the West Indies, Mount Hope, TTO; 2 School of Dentistry, Faculty of Medical Sciences, The University of the West Indies, Mount Hope, TTO; 3 Faculty of Medical Sciences, The University of the West Indies, Mount Hope, TTO; 4 Dean's Office, Faculty of Medical Sciences, The University of the West Indies, Mount Hope, TTO

**Keywords:** clinical training, covid-19, future performance, impact, learning behaviours, medical students, perceived stress

## Abstract

Introduction: The COVID-19 pandemic prompted the implementation of online education worldwide but the impact on medical student learning in the Caribbean is unclear.

Aim: The study aims to assess the impact of COVID-19 on medical students’ learning behaviours, clinical training, perception of performance as future medical professionals, and perceived stress.

Methods: A cross-sectional web survey on selected variables, utilising purposive sampling amongst all year four and year five undergraduate medical students in the University of the West Indies in Trinidad was conducted. Quantitative data was examined using χ2, Median (IQR) and qualitative information from open-ended questions was integrated with the presentation of the results.

Results: A total of 145 medical students responded of which 114 (78.6%, p<0.01) indicated that online learning strategies had brought a change in their learning behaviours and 136 (93.8%, p<0.01) respondents indicated that COVID-19 had affected the quality of their training, in most cases negatively. In the perception of future career performance, 90 (62.1%, p<0.01) respondents indicated that they felt a lack of preparation for working as a physician at the end of their training. It was found that 137 (94.5 %, p<0.01) fell into the moderate to high stress category.

Conclusions: COVID-19 had a significant impact on learning behaviours, clinical training and perception of performance in future career. Moderate to high levels of stress were observed among these students. These findings underscore the need for medical schools to adopt hybrid models that balance online education with practical clinical exposure to mitigate learning and psychological challenges experienced during the pandemic.

## Introduction

Coronavirus disease 2019 (COVID-19) forced changes to be made to curriculum delivery in order to accommodate continuity [1]. Although students were still at a disadvantage in some respects, for example, face-to-face interactions were not an option for long periods during the COVID-19 pandemic, there was however an opportunity for students to demonstrate their rapport-building skills in a new innovative way, allowing for students to learn how to adapt quickly to any situation they may encounter in their future careers. While virtual medical teaching during the COVID-19 pandemic had its positives, it also came with several negatives [2]. Despite the recognized impact of online learning in medical education, limited research has examined its effects on clinical training and mental health in Caribbean medical schools. This study seeks to address this gap by assessing the experiences of students at the University of the West Indies. Online teaching allowed for new interactive platforms and teaching between students and professors, at the same time allowing for students to keep up to date with new information via internet access. However, technical issues such as reduced engagement of students and also negative mental well-being, as well as lack of social interaction and in-person connection, are all weaknesses of virtual teaching and this may compromise the quality of learning for medical students. 

According to the WHO, the COVID-19 outbreak began in China in December 2019 and was declared a public health emergency on January 30th, 2020. On March 12th, 2020, Trinidad and Tobago reported its first confirmed COVID-19 case. Due to the high rate of transmission of the virus, one of the areas most affected is education as schools and universities were forced to close worldwide [3]. Closure of educational institutions led to a significant shift from face-to-face education to virtual learning, which negatively affected the quality of schooling for university students [4,5]. E-learning poses several disadvantages towards higher-level university students in particular. Their progression from the educational field to the job field is directly impaired by social distancing measures that prevent the practical application of their knowledge, reducing future career preparation. Undoubtedly, these steps adversely affected an integral part of medical education, that is, clinical training [6-8]. 

While a majority of students agree that online learning is both easily implemented and achievable, there was doubt that clinical skills would be sufficiently taught [9], and this could in turn affect the ability of students to obtain a holistic medical school experience and confidence in their skills. Not only has online teaching led to an increase in demands for clinical and administrative assistance from medical schools but it has undeniably challenged the medical educationists' ability to adjust the delivery of their syllabi to the current social restrictions [10,11]. E-learning has been shown to be an efficient modality of learning in various courses of study, where data reveals a more active attitude in learning when alternative methods are implemented [12-14]. In terms of medical education, this transition to digital schooling serves to limit clinical exposure and theory application of students who are in the penultimate and final years of study. Motilal et al. (2021) commented on digital schooling as follows: "The inability to reliably test physician skills at physical examinations on real patients was a limitation" [15].

As a result of a lack of clinical exposure and a drastic change from the typical learning environment, many students may have to adjust their learning behaviours to cope with the ‘new normal’ created by this pandemic. This has inadvertently created increased stress for medical students worldwide. Undergraduate medical students were found to have high levels of stress, depression and anxiety induced by COVID-19 in some studies [16-18] in India and China. Female medical students in Turkey compared to males experienced high levels of stress [19] but the majority of students who experienced severe stress were those during their transitional year in Saudi Arabia [20]. Whether the anxiety is due to the stress of online schooling, lack of preparedness, risk of contracting the virus or having a close family member contract it, students’ levels of anxiety are rising and should be carefully monitored [18]. Hence COVID-19 has not only taken its toll on the grades of medical students but also their mental health. It is not known whether the medical students in the University of the West Indies (UWI) in Trinidad are suffering the same levels of anxiety, stress or depression. This study seeks to evaluate the changes in medical students' learning behaviors, clinical training, perceived stress, and confidence in their future professional roles due to the shift to online education during COVID-19.

The UWI is a regional university that serves 15 countries with five campuses located in the Anglophone Caribbean. The UWI was founded in 1948 at Mona, Jamaica. It began first as a College of the University of London. In that year, 33 students from nine Caribbean countries were admitted to the founding Faculty of Medicine. In 1961, the UWI became an independent entity and about that time, it established two other campuses, first in Trinidad and Tobago at St. Augustine and later at Cave Hill, Barbados [21]. The UWI Faculty of Medical Sciences (FMS), St. Augustine campus (STA), Trinidad and Tobago began teaching its undergraduate Bachelor of Medicine, Bachelor of Surgery (MBBS) program in October 1989 and the first batch of medical students graduated in 1994. The faculty serves a key role in providing health professionals for the Caribbean region [22]. Further in the Caribbean, there is a scarcity of research on the impact of COVID-19 on medical education. Therefore, it is necessary to delve into the benefits or negative impacts of this unexpected shift in the delivery of clinical education that has occurred at the UWI STA with medical students. This will allow the provision of suggestions and improvements for similar scenarios as this may impact the future cohort of doctors within the Caribbean region and possibly the level of patient care. The aim of this study was to assess the impact of COVID-19 on medical students’ clinical training, learning behaviours, performance as future medical professionals, and perceived stress.

## Materials and methods

Ethical approval

The study was approved by the Campus Ethics Committee of the University of the West Indies (CREC-SA.0630/11/2020) and approval to conduct the study was received from the Campus Registrar. To realise the aims of the study, a cross-section web survey was conducted.

Data collection instruments 

The instrument consisted of five sections and was developed locally by adapting from previously published tools. Section A contained demographics and for Section B, the questionnaire used by Meo SA et al. (2020) was adapted by the authors in order to assess learning behaviours [23]. For Section C, the perceived stress scale developed by Cohen S (1994) [24] was used. The Perceived Stress Scale (PSS) is the most widely used psychological instrument for measuring the perception of stress. Sections D and E, clinical training and future career items, were developed by the authors using a wide range of literature sources and tacit knowledge, which was further reviewed by five clinical students to ascertain the suitability of the items for the variable they measure. Each section utilised a four-point Likert scale as well as open-ended questions, except the perceived stress scale section, to effectively measure the impact of COVID-19 on learning behaviours, clinical training, confidence in becoming a future clinician and perceived stress. The questionnaire is available in the Appendix.

Study population 

The target population consisted of 248 fourth-year and 212 fifth-year students at the University of the West Indies St. Augustine campus, Trinidad, pursuing an MBBS Degree in the Faculty of Medical Sciences. Year 4 and Year 5 are traditionally, before COVID-19, exposed to face-to-face clinical teaching as required by the five-year MBBS degree.

Study sample and size

This study utilised purposive sampling and targeted all clinical year fourth-year and fifth-year medical students pursuing a MBBS degree at the University of the West Indies, St. Augustine campus. The questionnaire was distributed to 460 medical students (248 fourth-year students and 212 fifth-year students).

Research approach and data collection process

The study was a cross-sectional web survey conducted from February to April, 2021. In distribution, a link to the questionnaire on Google Forms was sent to the UWI email ID of fourth-year and fifth-year medical students pursuing the MBBS degree at the University of the West Indies, St. Augustine. Google Forms were used to disseminate the questionnaire, allowing the students’ responses to be completely anonymous. As a result, participants were reassured of maximum confidentiality and privacy, for honesty and reliability. This link was re-forwarded as a reminder throughout the course of data collection as students did not respond immediately. At least four reminders were sent out via email at approximately three-week intervals.

Methods of data analysis

The data collected from the study were analysed using SPSS version 27 software (IBM Corp., Armonk, NY, USA). It was analysed through the use of a mean, standard deviation, percentages, with chi-square and median with interquartile range (IQR). The responses to open-ended questions in the impact on Learning Behaviour, Clinical Training and Future Career sections were integrated with quantitative findings to explain the phenomena in our quantitative data. The qualitative information generated through open-ended questions, except the Perceived Stress Scale section, were integrated with the quantitative findings. 

For psychometric properties of the instruments, the reliability coefficients established though Cronbach alpha values for Impact on Learning Behaviour (0.69), Clinical Training (0.66), Future Career (0.77) and Perceived stress (0.88) were found to be of acceptable levels [25].

## Results

Demographic distribution of the selected sample 

A total of 145 students responded: 98 (67.6%) respondents were Year 4 students while 47 (32.4%) were Year 5 students. The response rate for Year 4 students was 39.5% while that of Year 5 students was 22.2%. The sudden change of learning approach during the final year and less time to adjust to the learning environment might have resulted in low response among Year 5 students. Out of the total number of responses, 93 (64.1%) were female and 52 (35.9%) were male, which reflects the gender distribution in the University of the West Indies. The overall mean age was 24.37 ± 2.205. The mean age for Year 4 students was 23.92 ± 1.881, and for Year 5 was 25.32 ± 2.529. 

Impact on learning behaviours 

One hundred fourteen students (78.6%) agreed that online learning strategies have brought an overall change in their learning behaviour (median=3, IQR=0). One hundred five (72.4%) of respondents agreed that they noticed a deterioration in their study/work performance (median=3, IQR=1), 108 (74.5%) agreed that they experienced work burnout during the COVID-19 pandemic (median=3, IQR=2) compared to the prior situation and 107 (73.8%) expressed that their motivation level was affected due to not being amongst peers (median=3, IQR=1). Furthermore, 98 (67.9%) of respondents disagreed on appropriately concentrating on their studies (median=2, IQR=1) (section A of Table 1). 

**Table 1:** Impact of COVID-19 on learning behaviour, clinical training and future career of clinical year medical students

Questions	Strongly Disagree	Disagree	Agree	Strongly Agree	Chi Square	Median (IQR)	
A. Impact of COVID-19 on Learning Behaviour							
I am noticing a deterioration in my study/work performance.	12 (8.3%)	28 (19.3%)	80 (55.2%)	25 (17.2%)	74.393	3 (1)	
I am able to remember the contents of each online rotation (objectives, lecture notes and textbook material) appropriately.	27 (18.6%)	84 (57.9%)	29 (20.0%)	5 (3.4%)	93.648	2 (0)	
I am appropriately concentrating on my studies.	16 (11.0%)	82 (56.6%)	37 (25.5%)	10 (6.9%)	88.076	2 (1)	
My motivation level has been affected due to not being amongst my peers in the medical school environment.	8 (5.5%)	30 (20.7%)	76 (52.4%)	31 (21.4%)	67.441	3 (1)	
I have experienced work burnout during the COVID-19 pandemic compared to the prior situation.	7 (4.8%)	30 (20.7%)	67 (46.2%)	41 (28.3%)	51.386	3 (2)	
I am having difficulty in recalling recent information.	5 (3.4%)	36 (24.8%)	81 (55.9%)	23 (15.9%)	87.028	3 (1)	
I am having difficulty in recalling old information.	3 (2.1%)	24 (16.6%)	85 (58.6%)	33 (22.8%)	100.49	3 (0)	
I am studying for an increased number of hours.	15 (10.3%)	68 (46.9%)	41 (28.3%)	21 (14.5%)	47.303	2 (1)	
I am able to fully engage in clinical and critical thinking during online lectures and interactive sessions.	29 (20.0%)	82 (56.5%)	29 (20.0%)	5 (3.4%)	87.579	2 (0)	
I think that online learning strategies have brought an overall change in my learning behaviour.	7 (4.8%)	24 (16.6%)	87 (60.0%)	27 (18.6%)	101.152	3 (0)	
B. Impact of COVID-19 on clinical training						
If clinical training was done face to face, were there appropriate safety measures taken during this period (PPE, social distancing etc.)	17 (11.7%)	29 (20.0%)	86 (59.3%)	13 (9.0%)	94.862	3 (1)	
I have a dedicated space for online classes that is conducive to learning.	15 (10.3%)	30 (20.7%)	83 (57.2%)	17 (11.7%)	84.048	3 (1)	
I have easy access to devices (laptops, tablets, phones etc.) and/or stable internet connectivity.	3 (2.1%)	16 (11.0%)	76 (52.4%)	50 (34.5%)	90.614	3 (1)	
I am easily distracted during online classes.	4 (2.8%)	20 (13.8%)	74 (51.0%)	47 (32.4%)	78.476	3 (1)	
I think that the online clinical platform is as effective as face-to-face teaching.	48 (33.1%)	72 (49.7%)	21 (14.5%)	4 (2.8%)	74.172	2 (1)	
As a medical student, I support the measures proposed by the Ministry of Health on tertiary education institutions (closure of libraries, closure of schools, suspension of clinical training).	20 (13.8%)	25 (17.2%)	56 (38.6%)	44 (30.3%)	23.193	3 (2)	
I feel that I will be able to adequately reacquaint myself with all clinical learning aspects when face-to-face learning resumes.	21 (14.5%)	46 (31.7%)	60 (41.4%)	18 (12.4%)	33.786	3 (1)	
I feel that the lack of patient interaction associated with online learning, has affected my quality of learning.	4 (2.8%)	5 (3.4%)	70 (48.3%)	66 (45.5%)	111.469	3 (1)	
There was a lack of feedback on my medical skill performance as a result of online classes.	3 (2.1%)	16 (11.0%)	80 (55.2%)	46 (31.7%)	97.234	3 (1)	
The lack of use of clinical instruments on patients or simulated patients in online classes has affected my confidence in becoming a future medical professional.	8 (5.5%)	15 (10.3%)	38 (26.2%)	84 (57.9%)	97.455	4 (1)	
COVID-19 has affected the overall quality of my clinical training.	1 (0.7%)	8 (5.5%)	61 (42.1%)	75 (51.7%)	114.614	4 (1)	
C. Impact of COVID-19 on future career of clinical year medical students						
I feel at a disadvantage compared to prior years/seniors who were able to experience face-to-face clinical training for all of the program.	5 (3.4%)	15 (10.3%)	40 (27.6%)	85 (58.6%)	105.345	4 (1)	
Online clinical learning has positively affected my interest in becoming a medical doctor.	24 (16.6%)	91 (62.8%)	26 (17.9%)	4 (2.8%)	118.421	2 (0)	
I believe that this pandemic experience will be beneficial to my future career in medicine.	12 (8.3%)	29 (20.0%)	89 (61.4%)	15 (10.3%)	106.89	3 (1)	
Online classes have affected my communication skills.	11 (7.6%)	41 (28.3%)	62 (42.8%)	31 (21.4%)	37.262	3 (1)	
Online classes have affected my familiarity to work in a hospital as a future medical professional.	4 (2.8%)	27 (18.6%)	72 (49.7%)	42 (29.0%)	67.221	3 (1)	
I feel that I will be prepared to work as a future medical professional in a hospital at the end of my training.	23 (15.9%)	67 (46.2%)	42 (29.0%)	13 (9.0%)	46.752	2 (1)	
I feel confident in performing a clinical examination.	35 (24.1%)	58 (40.0%)	43 (29.7%)	9 (6.2%)	34.834	2 (1)	
Online classes have affected my confidence in having doctor-doctor and patient-doctor interactions.	5 (3.4%)	34 (23.4%)	73 (50.3%)	33 (22.8)	64.628	3 (1)	
The social stigmatization of graduating from medical school during the pandemic will affect my future career in healthcare.	12 (8.3%)	35 (24.1%)	66 (45.5%)	32 (22.1%)	41.179	3 (1)	
Online classes have affected my team-based learning, patient interviewing skills and physical exam performance.	6 (4.1%)	25 (17.2%)	77 (53.1%)	37 (25.5%)	74.559	3 (1)	
I have been able to explore alternative means of patient interaction (apart from face-to-face) due to the constraints of learning during a pandemic.	24 (16.6%)	48 (33.1%)	58 (40.0%)	15 (10.3%)	33.455	3 (1)	
I feel deprived of learning proper physical examination skills due to lack of face-to-face clinical training.	4 (2.8%)	15 (10.3%)	47 (32.4%)	79 (54.5%)	94.752	4 (1)	
Learning during a pandemic has better prepared me for practicing medicine during resource- challenging times, and managing complex patients in a high-stake setting.	24 (16.6%)	53 (36.6%)	31 (21.4%)	37 (25.5%)	12.655	2 (2)	
The COVID-19 pandemic has affected my choice of specialty in the field of medicine.	39 (26.9%)	69 (47.6%)	23 (15.9%)	14 (9.7%)	48.297	2 (2)	
Courses within my programme have adequately prepared me for working in a hospital during a pandemic.	30 (20.7%)	43 (29.7%)	59 (40.7%)	13 (9.0%)	31.524	2 (1)	

Responding to the open-ended question in this section about "List any other changes in your learning behaviour during COVID-19" only 109 (75.2%) out of 145 recorded their responses. Firstly, many students expressed increased feelings of demotivation. Some respondents claimed that their learning behaviour changed because of the "lack of motivation and focus due to being at home" while others linked their declining mental health to their decreased motivation to study, many noting increased "burn-out." Students reported decreased concentration, indicating a "shortened attention span" due to increased time at home. One respondent noted, "The increased screen time has caused severe headaches thus impacting my ability to concentrate during the lecture."

One respondent acknowledged this, stating, "Helpful group study sessions are now limited, diminishing the learning experience brought about by the pooling of information and asking each other questions." Both Year 4 and Year 5 medical students indicated that their learning behaviour during the pandemic changed because of a lack of motivation, which created difficulties when concentrating and ultimately led to a modified study pattern that consisted of less studying and a lack of structure. Respondents articulated, "More hours online results in great mental fatigue, hence less study time."

Students reported changed study patterns, expressing that they were studying for a reduced number of hours. Also 83 (57.2%) agreed that they were not studying for an increased number of hours (median=2, IQR=1). Lastly, another major change in learning behaviour noted by students was an increased dependence on online resources with students claiming, "I have an increased reliance on online tools e.g. YouTube." E-Learning created a high dependence on online resources and tools, which increased their screen time contributing to headaches and eye-pain that hindered their concentration. One student stated, "It is difficult to learn online, I have been having very bad headaches and pain in my eyes as well, most likely due to focusing on a screen for most of the day."

Impact on clinical training 

One hundred thirty-six (93.8%) respondents agreed that the lack of patient interaction associated with online learning has affected their quality of learning (median=3, IQR=1) (Table 1, section B). This lack of patient interaction perception issue is further echoed while 135 (93%) out of 145 students responded for the first open-ended question about means and ways of improving delivery of online clinical learning. Many students requested the use of simulated patients and rounds, video compilations of clinical scenarios commonly seen on the ward, as well as case-based learning. One student suggested, "To improve it, I believe the lecturers can try to make the classes as clinical as possible. Give case scenarios of real patients and how to diagnose and treat etc. rather than just covering theory." Additionally, students advocated for interactive small group discussion with respondents requesting, "smaller groups of people, so we can get a bit more individualised critique on our clinical skills instead of larger classes and a lecturer." Many linked large group sessions to a lack of feedback which explains why 126 (86.9%) of respondents noted a lack of feedback on their medical skill performance as a result of online classes (median=3, IQR=1).

One hundred thirteen (77.9%) out of 145 students responded to the open-ended question regarding advantages attached with online clinical training. Most students noted increased time for relaxation while others noted that online classes were convenient as it allowed for students to remain at home, reducing travel expenses and increasing comfort.

One hundred twenty-two (84.1%) respondents agreed that the lack of use of clinical instruments on patients or simulated patients in online classes has affected their confidence in becoming a future medical professional (median=4, IQR=1) (Table 1, section B). The preceded finding is further reflected in 123 (85%) out of 145 students who responded to the open-ended question on the challenges associated with online learning. Most of the respondents expressed a lack of patient and ward experience with one student explaining, "I feel a distancing from my confidence in practical skills as the online experience prevents adequate real-life practice." For many students the home environment also created difficulties during this period of online learning. One student argued, "internet connection / electricity difficulties; noise of relatives while trying to study; perceived by family members as 'available', when in fact you have much to do (generally: clash of family dynamic with school dynamic - messy, disorganised, frustrating)." This can be linked to 121 (83.4%) respondents indicating that they were easily distracted during online classes (median=3, IQR=1). When questioned regarding the efficacy of online clinical platforms, 120 (82.8%) respondents stated that the online clinical platform is not as effective as face-to-face teaching (median=4, IQR=1). One respondent expressed that "At this point, I think regardless of improvements, online training cannot be a substitute for hands-on training. Many online rotations were well done and lecturers implemented history taking and video sessions to simulate a surgery viewing and other innovative methods, but the fact is, it cannot make up for soft skills like learning to navigate the ward environment, communication with nurses and other staff as well as the necessary clinical skills."

Impact on performance as future medical professionals 

One hundred four (71.7%) respondents indicated that they feel as though this pandemic experience would be beneficial to their future career (median=3, IQR=1) (Table 1, section C). One hundred four (71.7%) out of 145 students who responded to the open-ended question, "List one aspect of online learning you believe will positively impact you as a future medical doctor," wherein knowledge, adaption and face-to-face appreciation were three major highlighted responses. Regarding knowledge, respondents stated that they have acquired a greater level of theoretical knowledge due to the increased amount of time they are able to spend on this section of the curriculum. One student mentioned, "Since all classes were online, a lot of theory was covered that would have otherwise been self-directed learning. This gave us an advantage of knowing a lot more than previous years before we are sent out on the wards."

Many students claimed that online learning allowed them to gain the skill of adaptation. This explains why 73 (50.3%) respondents agreed that they have been able to explore alternative means of patient interaction (apart from face-to-face) due to the constraints of learning during a pandemic (median=3, IQR=1). One student stated that, "I was able to adapt to adverse, unexpected circumstances" while another articulated that online learning, "Opened up telemedicine opportunities." One hundred twenty-six (86.9%) respondents indicated that they feel deprived of learning appropriate physical examination skills due to lack of face-to-face clinical training (median=4, IQR=1). One student noted, "I developed a greater appreciation for face-to-face classes" while another stated, "Greater appreciation for the school environment."

Impact on Perceived Stress Scale (PSS)

The PSS female mean score was 23.68 ± 6.12, which was higher than the male mean score of 22.58 ± 6.97, but the difference was not statistically significant. The PSS mean score for Year 5 was 24.04 ± 6.16, which was higher than the Year 4 students of 22.92 ± 6.57, but statistically insignificant. The overall PSS mean score was 23.28 ± 6.43. 

However, a chi-square test of equality for perceived stress levels showed a significant difference in different levels of perceived stress, χ2 (2, N=145)=86.497, p < .01. A significant percentage of students experienced moderate stress to high stress during the period (Table 2).

**Table 2:** Table showing the Perceived Stress Scale (PSS) chi-square test of equality

	Low (0-13)	Moderate (14-26)	High (27-40)	Chi square	P value
PSS score	8 (5.5%)	98 (67.6%)	39 (26.9%)	86.497	.01

## Discussion

The study received 145 responses and the results displayed profound changes in the learning behaviours of clinical year students, moderate levels of stress, a considerable decline in the quality of clinical education and training and reduced confidence in performance as future medical professionals. Additionally, responses revealed a need for the online delivery of clinical education to be redesigned to increase its efficiency, with students proposing beneficial solutions and suggestions in the qualitative sections of the questionnaire distributed to them. Moreover, this study was instrumental in displaying the negative effects of the COVID-19 pandemic on students’ attitudes and behaviours and prompted questions on how these difficulties could be eliminated in the future. Evidently, results from this study may guide the improvement of the online delivery of medical education in the Caribbean.

The analysis of the qualitative questions revealed four major concerns for change in learning behavior: lack of motivation, dependence on online resources, changed study pattern and lack of concentration. Five areas emerged for online clinical learning to be improved: use of audio-visual aides, clinical scenarios, interactive small group discussions, face-to-face teaching and organisation. The transition to online from the wards has resulted in two advantages for students: time and convenience but while listing disadvantages it included lack of patient and ward experience, lack of motivation and an unconducive environment. Other areas about what will positively impact their future as a medical doctor were knowledge, adaptation, appreciation of face-to-face interaction and use of telemedicine.

In view of the COVID-19 pandemic and quarantine phase, medical students were forced to suddenly divert from face-to-face learning to E-learning. It has been shown that technology for online learning can be harnessed to facilitate medical instruction and courses emphasising skills such as open communication and medical ethics [26]. Although many medical students in this study support the measures proposed by the Ministry of Health on tertiary education institutions closure such as libraries, schools and suspension of clinical training for online learning, this abrupt shift has affected the learning behaviour of clinical year medical students considerably, exposing altered habits and study patterns.

Our study was similar to another study [27] that found most medical students had a positive perception of E-learning and the majority of students agreed that the closure of the university is an essential decision to control the spread of the COVID-19 infection [28]. However, there are many challenges considered as inhibitory factors for utilising electronic technologies for medical education. In our study, for instance, 114 (78.6%) of respondents agreed that online learning strategies brought an overall change in their learning behaviour and 105 (72.4%) of those respondents noticed a deterioration in their study/work performance. Our findings found that both female and male medical students identified a decrease in their overall work performance, which is consistent with Meo et al. (2020) [23] and also a decrease in time they spent studying [28]. Moreover, students emphasised an inability to have a structured day after the shift to online learning.

In addition, our study found that 76.5% of the students disagreed with being fully engaged in clinical and critical thinking during online lectures and interactive sessions due to the lack of motivation. This is supported by findings elsewhere that indicated the use of online materials and platforms is often extensively utilised by universities and other educational institutions during the pandemic [29]. It has its limitations in which a key element of medical training is developing interpersonal and practical skills, something that is learnt through on-site clinical attachments. Therefore, digital learning is not a sufficient substitute for acquiring these attributes fundamental to being a doctor.

The current COVID-19 pandemic poses a unique set of circumstances that have potential to further affect stress levels, leading to physical and mental health decline. Surveys carried out so far found that 96 (48%) of respondents overall experienced pandemic-related anxiety and stress [30] and that COVID-19 had contributed to poor mental health [31]. Our study found that the majority, 98 (67.6%), of Year 4 and Year 5 students experienced moderate stress, followed by 39 (26.9%) who experienced high stress and lastly eight (5.5%) who experienced low stress. Thus, our findings mirrored several others [8,17,20,30-32]. However, one study [33] in the United Arab Emirates noted that the introduction of online classes surprisingly decreased the level of anxiety among some medical students in clinical rotations. Therefore, the impact of the COVID-19 pandemic on perceived stress among medical students seems to vary within its diverse aspects and is highly influenced by a student’s support system and their physical environment. Differences in stress levels amongst males and females were not significant in this study, however other studies show mixed findings where some found that males had statistically significantly lower stress levels than females [34] while others found that female students had higher stress levels than males [35].

One hundred twenty (82.8%) students who participated in this study disagreed that the online clinical platform is as effective as face-to-face teaching and this was in agreement with the findings of Syed et al. (2021) [36]. This was also reflected in the open-ended questions where students declared that there was no substitute for face-to-face learning. When asked how the delivery of online learning could be improved, students simply explained, "It can't, online learning is not an effective method for training in a practice-based career."

One hundred twenty-two (84.1%) participants in this study indicated that the lack of use of clinical instruments on patients or simulated patients in online classes affected confidence in becoming a future medical professional and this is in agreement with the findings of others [37-39]. Students mentioned that the large group settings for online classes prevented one-on-one interactions and established a hostile environment for discussion as question-and-answer sessions involved public speaking which created discomfort. Respondents in this study advocated for the implementation of audio-visual aids and increased clinical application and this was the case in another study [40]. On a positive note, however, when students were asked about the advantages of the online platform, students indicated that online learning afforded them more time for relaxation and was more convenient due to the comfort of the home environment, consistent with some previous studies [41,42]. The sample of students who stated that they had easy access to devices (laptops, tablets, phones etc.) and/or stable internet connectivity in this study was 126 (86.9%) compared to 19 (13.1%) who did not. Although only 19 (13.1%) of respondents experienced difficulty accessing devices, this can have adverse effects on their online learning experience [43,44].

This study showed that 90 (62.1%) respondents indicated that they do not feel prepared to work in the future, while 93 (64.1%) indicated that they were not confident. This has also been observed in other studies that medical students did not wish to return to the clinical setting for fear of putting patients at risk due to their lack of training [45-47]. Additionally, Sharma and Bhaskar (2020) [48] found that students were "Lost in-hospital clinical clerkships which caused fears surrounding deficiency in practical skills, training and ‘imposter syndrome.’" It has been reported that students experienced fears around surgery preparedness due to lack of training [49-52]. In our study, 77 (53.1%) agreed that learning during a pandemic was beneficial, along with 108 (74.5%) indicating that their choice of specialty was unaffected; however, this was found to be the case in other studies [53-55].

One student mentioned a major advantage of online learning that would positively impact them as a future doctor, "Having online classes with seniors who I would not have come into contact with otherwise and gaining valuable knowledge." Nonetheless, students continued to acknowledge a newfound appreciation for face-to-face learning [56-58]. Respondents in Al-Balas et al. (2020) [59] also expressed that a blended approach which utilised both traditional and E-learning should be implemented in future medical training. Additionally, students articulated that adaptation proved to be a major positive impact of online learning. Students described an "increased ability to adjust to adverse and unexpected circumstances i.e. thinking on feet and finding solutions; critical thinking" which suggests that students have adapted to the pandemic situation by indulging in a variety of personal and professional endeavours, for example, research.

Online learning is not as effective as face-to-face learning and revisions and modifications to the delivery of online learning are necessary to facilitate better clinical education during a pandemic. Online courses should be restructured, placing student needs at the forefront and academic support systems enhanced. It is evident that there is a considerable negative impact on the learning behaviours of medical students in the UWI coupled with a deterioration in their study performance irrespective of gender and years of study. Most clinical medical students in Trinidad and Tobago believe that the transition to online learning during the COVID-19 period has negatively affected the quality of their clinical training. Although most students are equipped with devices to participate in online learning, there is a strong lack of human connection whether it be with instructors or lack of patient interaction that has negatively affected the clinical training which is of utmost importance in a practice-based field such as medicine. Overall, the vast majority of both Year 4 and Year 5 medical students in UWI are experiencing moderate stress levels, which may be linked to the mental and emotional stresses posed by the global pandemic, in addition to academic stress experienced in their clinical years. Lack of communication with instructors can lead to a decline in the supportive framework of the medical school environment.

Limitations of the study

The study was conducted at one medical school at one site and therefore the findings may not be generalisable outside of this medical school due to differences in the educational program structure. As this study relied on self-reported data, social desirability bias may have influenced responses. Additionally, findings may not be generalisable beyond the University of the West Indies due to differences in medical curricula across regions. The low response rate among final-year students may reflect their higher academic workload and stress levels, potentially skewing the data toward the experiences of younger students. This study had limitations such as non-response bias as many students did not respond to the survey and can affect the generalisability of the study. Also, self-reported data has its own limitation.

## Conclusions

Most clinical medical students in Trinidad and Tobago believe that the transition to online learning during the COVID- 19 period has negatively affected the quality of their clinical training and that there has been a considerable negative impact on their learning behaviours, coupled with a deterioration in their study performance irrespective of gender and years of study. 

Overall, the vast majority of both Year 4 and Year 5 medical students in UWI are experiencing moderate stress levels which may be linked to the mental and emotional stresses posed by the global pandemic, in addition to academic stress experienced in their clinical years. Additionally, medical students perceive COVID-19 to be a factor that has negatively impacted their future career as there was a lack of confidence due to social restrictions along with social stigmatisation of graduating from medical school during the pandemic. 

The findings of the study may be used by the university administrators to improve mental health support strategies as well as to encourage investment in simulation-based technologies to enhance medical education. These findings underscore the need for medical schools to adopt hybrid models that balance online education with practical clinical exposure to mitigate learning and psychological challenges experienced during the pandemic. While online learning posed significant challenges, some students reported benefits such as greater exposure to telemedicine and flexible study schedules, suggesting potential areas for future curriculum adaptation. Future research should explore hybrid training models incorporating telemedicine and augmented reality-based simulations to enhance clinical training during periods of restricted patient access.

## Appendices

Legend: Sections A-E

Credits: Sections A, D & E of the questionnaire were self-developed by the authors Bidyadhar Sa, Asilah Ali, Aviel Smith, Avonell Wickham, Bakhita Johnston, Betricia Stowe, Brandon Cross, Brandon Seenath, Brent Rajkumar, Section B adapted by the authors above from Meo SA, Abukhalaf AA, Alomar AA, et al. 2020 [23] and Section C, the Perceived Stress Scale (PSS) developed by Cohen S, 1994 [24]. The PSS is the most widely used psychological instrument for measuring the perception of stress. It is available free for not-for-profit research at https://eprovide.mapi-trust.org/instruments/perceived-stress-scale-10-items.

QUESTIONNAIRE Sections A-E

Impact of COVID-19 on medical students’ clinical training, their learning behaviours, perceived stress and performance as future medical professionals.

A.   BACKGROUND DEMOGRAPHICS:

Please fill in the following (items 1-4) before answering the questions provided:

Gender:

⬜Male                           ⬜Female              ⬜Other             

Age:

Year Group:

⬜Year 4                          ⬜Year 5

Ethnicity:

B.

**Table 3:** Section B- Impact on Learning behavior Section B adapted by the authors above from Meo SA, Abukhalaf AA, Alomar AA, et al. 2020 [23]

Section B.	IMPACT ON LEARNING BEHAVIOR:	Strongly Disagree	Disagree	Agree	Strongly Agree
1	I am noticing a deterioration in my study/work performance.				
2	I am able to remember the contents of each online rotation (objectives, lecture notes and textbook material) appropriately.				
3	I am appropriately concentrating on my studies.				
4	My motivation level has been affected due to not being amongst my peers in the medical school environment.				
5	I have experienced work burnout during the COVID 19 pandemic compared to the prior situation.				
6	I am having difficulty in recalling recent information.				
7	I am having difficulty in recalling old information.				
8	I am studying for an increased number of hours.				
9	I am able to fully engage in clinical and critical thinking during online lectures and interactive sessions.				
10	I think that online learning strategies have brought an overall change in my learning behavior.				

          11. List any other changes in your learning behaviour during COVID-19.

______________________________________________________________________

C.

**Table 4:** Section C- Perceived Stress Scale (PSS) Section C, the Perceived Stress Scale developed by Cohen S, 1994 [24]. The Perceived Stress Scale (PSS) is the most widely used psychological instrument for measuring the perception of stress. It is available free for not for profit research at https://eprovide.mapi-trust.org/instruments/perceived-stress-scale-10-items.

C. Section C.	PERCEIVED STRESS SCALE:	Never	Almost Never	Sometimes	Fairly Often	Very Often
1.	In the last month, how often have you been upset because of something that happened unexpectedly?					
2.	In the last month, how often have you felt that you were unable to control the important things in your life?					
3.	In the last month, how often have you felt nervous and “stressed”?					
4.	In the last month, how often have you felt confident about your ability to handle your personal problems?					
5.	In the last month, how often have you felt that things were going your way?					
6.	In the last month, how often have you found that you could not cope with all the things that you had to do?					
7.	In the last month, how often have you been able to control irritations in your life?					
8.	In the last month, how often have you felt that you were on top of things?					
9.	In the last month, how often have you been angered because of things that were outside of your control?					
10.	In the last month, how often have you felt difficulties were piling up so high that you could not overcome them?					

D. IMPACT ON CLINICAL TRAINING:

In an attempt to minimize the spread of the COVID-19 virus, adjustments have been made to the delivery of clinical education. How are you receiving clinical training during the COVID period? (please select one option)

 ⬜ Online

 ⬜ Face to face

 ⬜ Blended

**Table 5:** Section D - Impact on Clinical Training Credits: Sections A, D & E of the questionnaire were self-developed by the authors Bidyadhar Sa, Asilah Ali, Aviel Smith, Avonell Wickham, Bakhita Johnston, Betricia Stowe, Brandon Cross, Brandon Seenath, Brent Rajkumar

Section D.	IMPACT ON CLINICAL TRAINING:	Strongly Disagree	Disagree	Agree	Strongly Agree
1	If clinical training was done face to face, were there appropriate safety measures taken during this period (PPE, social distancing etc.)				
2	I have a dedicated space for online classes that is conducive to learning.				
3	I have easy access to devices (laptops, tablets, phones etc.) and/or stable internet connectivity.				
4	I am easily distracted during online classes.				
5	I think that the online clinical platform is as effective as face-to-face teaching.				
6	As a medical student, I support the measures proposed by the Ministry of Health on tertiary education institutions (closure of libraries, closure of schools, suspension of clinical training).				
7	I feel that I will be able to adequately reacquaint myself with all clinical learning aspects when face-to-face learning resumes.				
8	I feel that the lack of patient interaction associated with online learning, has affected my quality of learning.				
9	There was a lack of feedback on my medical skill performance as a result of online classes.				
10	The lack of use of clinical instruments on patients or simulated patients in online classes has affected my confidence in becoming a future medical professional.				
11	COVID-19 has affected the overall quality of my clinical training.				

12. How could the delivery of online clinical learning be improved?

13. List the challenges you experienced while transitioning to online learning from the ward dynamic.

14. List the advantages you experienced while transitioning to online learning from the ward dynamic.

E.

**Table 6:** Section E- Impact on Future Career Credits: Sections A, D & E of the questionnaire were self-developed by the authors Bidyadhar Sa, Asilah Ali, Aviel Smith, Avonell Wickham, Bakhita Johnston, Betricia Stowe, Brandon Cross, Brandon Seenath, Brent Rajkumar,

Section E.	IMPACT ON FUTURE CAREER:	Strongly Disagree	Disagree	Agree	Strongly Agree
1	I feel at a disadvantage compared to prior years/seniors who were able to experience face to face clinical training for all of the programme.				
2	Online clinical learning has positively affected my interest in becoming a medical doctor.				
3	I believe that this pandemic experience will be beneficial to my future career in medicine.				
4	Online classes have affected my communication skills.				
5	Online classes have affected my familiarity to work in a hospital as a future medical professional.				
6	I feel that I will be prepared to work as a future medical professional in a hospital at the end of my training.				
7	I feel confident in performing a clinical examination.				
8	Online classes have affected my confidence in having doctor-doctor and patient-doctor interactions.				
9	The social stigmatization of graduating from medical school during the pandemic will affect my future career in healthcare.				
10	Online classes have affected my team-based learning, patient interviewing skills and physical exam performance.				
11	I have been able to explore alternative means of patient interaction (apart from face to face) due to the constraints of learning during a pandemic.				
12	I feel deprived of learning proper physical examination skills due to lack of face-to-face clinical training.				
13	Learning during a pandemic has better prepared me for practicing medicine during resource- challenging times, and managing complex patients in a high- stake setting.				
14	The COVID-19 pandemic has affected my choice of specialty in the field of medicine.				
15	Courses within my programme have adequately prepared me for working in a hospital during a pandemic.				

16 List one aspect of online learning you believe will positively impact you as a future medical doctor.

______________________________________________________________________________

Thank you! 😃
